# Analysis of Massaciuccoli Peat after Maturation in Sodium Chloride Water of Undulna Thermae

**DOI:** 10.3390/ijerph19042169

**Published:** 2022-02-15

**Authors:** Laura Giuseppina Di Pasqua, Clarissa Berardo, Lorenzo Raffo, Andrea Ferrigno, Enrico Guffanti, Mariapia Vairetti

**Affiliations:** 1 Unit of Cellular and Molecular Pharmacology and Toxicology, Department of Internal Medicine and Therapeutics, University of Pavia, 27100 Pavia, Italy; lauragiuseppin.dipasqua01@universitadipavia.it (L.G.D.P.); clarissa.berardo01@universitadipavia.it (C.B.); andrea.ferrigno@unipv.it (A.F.); 2 Undulna Thermae, 54038 Cinquale, Italy; lorenzo.raffo@gmail.com

**Keywords:** peats, maturation, protein

## Abstract

In Italy, peat extracted from the peat bogs of Lake Massaciuccoli is the only peat used for therapeutic purposes. Massaciuccoli peat (M-peat) soaked in the salty bromine–iodine water of Undulna Thermae has given positive results in various pathological situations, mainly in dermatological, rheumatological, and traumatological conditions. Morphological and biochemical analysis were performed using base M-peat samples matured in the salty bromine–iodine water of the Undulna Thermae for different times, to evaluate whether maturation time modifies peat chemico-physical properties. The maturation process induced particle aggregation, with an increase in the fractions with larger particle size. The presence of a high number of proteins derived from organic degradation was observed; after 6 months of maturation, a significant increase in proteins was found, suggesting that salty bromine–iodine water plays a role in the clinical action of the peat. The presence of lipids in M-peat was also confirmed, allowing us to draw important considerations on its therapeutic properties possibly deriving from the relevant interactions between lipids and humic acids. Finally, from our observations, it could be reasonably argued that longer periods of maturation do not result in additional advantages regarding clinical activity.

## 1. Introduction

Balneo-mud therapy or peloidotherapy is a clinically effective complementary approach in the treatment of low-grade inflammation and stress-related pathologies. In spas (salus per aquam), the material derived from the maturation of mud, silts, molds, or peat with the specific water of the spa, is frequently used [1]. In Italy, the most used peloids are certainly mud; in Germany, Ukraine, and Russia, the use of peat is equally widespread. Peat undoubtedly represents the most important group among organic peloids. Peat can be thousands of years old; the processes of humification and demolition by the bacterial flora transform the vegetable organic mass into a homogeneous greasy substance without any residual structure. These are certainly the most valuable peats; the one used in the Undulna spa in Tuscany, located in central Italy, belongs to this category and is collected from Lake Massaciuccoli [2,3].

Peat is an organic compound that comes from the slow maceration of herbs and plants deposited in particularly wet and marshy environments. The plants, after long chemical and biological transformations, turn into a precious and vital living mixture, highly rich in minerals, enzymes, macromolecules (such as proteins, lipids, and cellulose), and decomposition products (such as humic acid, umolignin, humine, amino acids, and bitumen). These compounds have multiple biological activities, such as antibacterial, antifungal, immunomodulatory, and photoprotective activity, relevant for the use of peat in dermatology and cosmetics [4,5,6,7].

The use of Massaciuccoli peat (M-peat) for therapeutic purposes is effective in various pathological situations, mainly dermatological, rheumatological, and traumatological conditions [8,9,10,11].

The biological mechanisms by which immersion in mineral–medicinal water and the application of mud/peat alleviate symptoms of several pathologies are still not completely understood. To evaluate whether maturation time affects the chemical–physical qualities of M-peat, and to identify the peat with the correct maturation, an analysis was carried out on peat samples matured in the water of the Undulna spa (salty bromine–iodine) for different times. In particular, changes in total proteins, thiol groups, and total lipids were quantified, and morphological analysis was performed, in order to understand the mechanisms of action of this peat used in Italy for therapeutic purposes.

## 2. Materials and Methods

### 2.1. Materials

The peat collected from a peat bog of Lake Massaciuccoli was matured in suitable metal tanks with the salty bromine–iodine water that flowed from the subsoil at 17 °C (Table 1). The ash in the M-peat was 14.58% [8].

The base M-peat remained immersed in thermal water for different maturation periods and, in any case, not less than six months. After maturation, M-peat was placed in special containers ready to be applied to patients. The soaking process was carried out in a mineral water tank to which suitable quantities of peaty material were added (from a few grams up to 50 kg and more). The final temperature used was usually lower than that of a common bath and it was set up between 34 and 35 °C, similar to that used for sulfurous and carbonic water baths.

Four M-peat samples were examined:

- Basic peat before being introduced into the maturation cycle;
- Peat matured for 6 months;
- Peat matured for 16 months;
- Peat matured for 36 months and ready to use on the patient.

In the peat samples, the water content was around 54% and solids around 46%. No significant difference occurred in the four peat samples considered.

### 2.2. Morphological and Biochemical Analysis

The morphological analysis of peloids was performed previously [12]. In the present study, Zoe Cell Imager (Bio-Rad) was used for peat samples (5 mg) resuspended in 0.5 mL of distiller water.

For the extraction process, 20 mL buffer (1.5 M Tris HCl, 0.4% SDS, 8 mM EDTA, pH 8.8) was added to 10 g of peat. Samples were mixed for 45 min at room temperature and then centrifuged for 10 min at 4000× *g*. The supernatants were collected for the analysis of total protein, thiol protein, and lipid content.

Total protein content was determined in accordance with the Lowry method using albumin as standard [13].

The concentration of thiol protein groups (protein-SH) was estimated using 5V-5V-dithio-bis(2-nitrobenzoic acid) (DTNB), as described by Di Monte et al. [14].

Nile Red was used for lipid detection and its fluorescence was measured using a Victor^2^ Multilabel Counter Wallac (Perkin Elmer) [15]. Samples for lipid detection were prepared as follows: 5 μL sample and 5 μL Nile Red 1mg/mL were added to 190 μL of PBS buffer (sodium phosphate dibasic anhydrous 8 mM, sodium dihydrogen phosphate monohydrate 2 mM, 140 mM sodium chloride, pH 7.4). After incubation at room temperature (10 min), samples were loaded on a 96-well plate and read at 485 nm excitation and 572 nm emission.

### 2.3. Statistical Analysis

The statistical analysis was carried out using MedCalc Statistical Software version 18.11.3 (MedCalc Software bvba, Ostend, Belgium; https://www.medcalc.org; 2019). Normal data distribution was analyzed by Kolmogorov–Shapiro normality test. Statistical analysis was performed with one-way ANOVA, followed by Tukey’s multiple comparisons test.

## 3. Results

### 3.1. Morphological Analysis

From visual analysis, the color of the peat samples did not differ when comparing the different stages of maturation.

**Figure 1:**
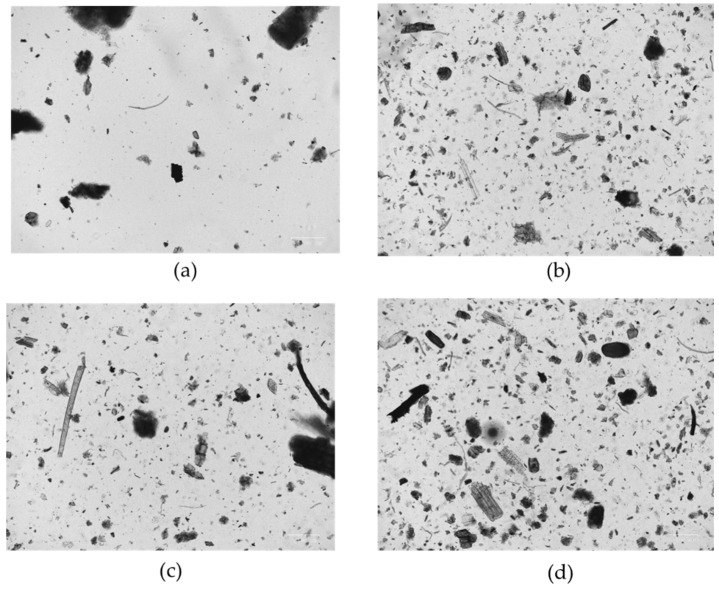
Microscopic analysis of M-peats: basic peat (**a**), after 6 months (**b**), after 16 months (**c**), and after 36 months (**d**) at 20× magnification.

Microscopic analysis (20× magnification) showed differences in the distribution of particle size in relation to different maturation times, when compared to the basic pre-maturation peat. The maturation process with salty bromine–iodine water involved a tendency towards particle aggregation, with an increase in the fractions with larger particle size (Figure 1). This phenomenon was already appreciable after 6 months of maturation. The formation of macroaggregates tended to increase with the duration of maturation.

### 3.2. Total Protein, Thiol Protein, and Lipid Content in Peat in Relation to the Maturation Period

**Figure 2:**
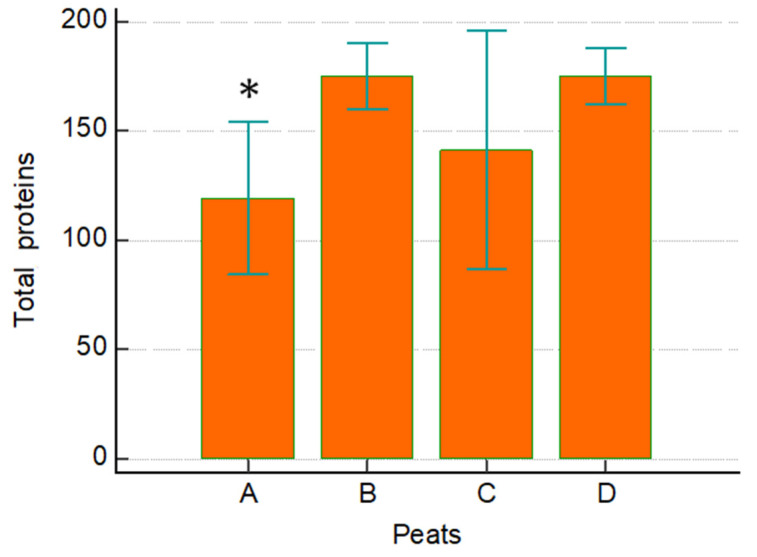
Number of proteins (mg/mL) found in the M-peat samples: basic peat (A), after 6 months (B), after 16 months (C), and after 36 months (D). * *p* = 0.002 versus B and D.

The average level of protein content found in the M-peat was significantly higher after 6 months and 36 months of maturation with salty bromine–iodine water from the spa, in comparison to the protein content found in the base peat. No significant differences were observed between the base peat and the 16-month-matured peat, as well as between the three different maturation times (Figure 2 and Table 2).

**Figure 3:**
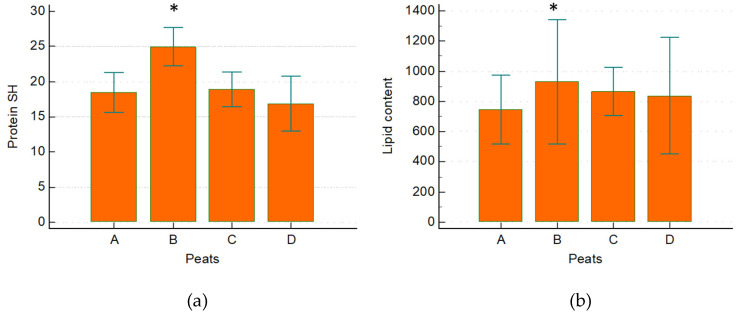
Number of proteins SH (µM) (**a**) and lipid content (A.U.) (**b**) found in the M-peat samples: basic peat (A), after 6 months (B), after 16 months (C), and after 36 months (D). Protein SH: * *p* < 0.001 versus A, C, and D. Lipid content: * *p* = 0.026 versus A.

Low levels of SH groups were present in all peat samples at the different maturation times; a significant difference was found when comparing the 6-month-matured peat to the other peats (Figure 3 and Table 2).

The presence of lipids was observed in all peat samples; significantly higher levels were observed in the 6-month-matured sample (Figure 3 and Table 2).

## 4. Discussion

### 4.1. Components of the Peat

With regard to the macroscopic and microscopic aspect, it should be remembered that, depending on the type of peat, there are different degrees of decomposition, related to different factors that come into play in this process. Lower levels of decomposition are typical of young peat, while higher levels are typical of more mature and unstable peat, as is evidently the case with the M-peat.

The differences in granulation at different times of maturation are probably due to different degrees of decomposition related to the time of contact with the salty bromine–iodine water of the spa. Unlike what has been observed in some muds, the finer granulometry increases at different times of maturation. In addition, the application of mud with incorrect maturation does not generate an increase in beta endorphin and ACTH typically observed between 2 and 10 min after the application of 5–6-month-matured mud [16].

Peat is made up of 90% water, humic acids, fulvic acids, humins, pectins, wood, cellulose, waxes, resins, and inorganic material. Therefore, it is not surprising to observe proteins in the samples brought to examination. Some consideration should be given, however, to the trend in the different sample with different maturation times. It is evident that the increase in protein content, already evident and significant at 6 months, comes from the maturation with the thermal water. These levels are similar to those at 36 months; the lower quantity found in the sample at 16 months vs. 6 and 36 months could provide explanations of a technical conservative nature and still needs further confirmation in order for us to better understand the real effectiveness of the maturation processes of an organic material such as peat. It should be noted, however, that the number of proteins observed in peat at 16 months is still greater than that of base peat, although not reaching levels of statistical significance.

The presence of a high amount of protein would further confirm the known diversity between organic and inorganic materials [17] and all that this entails in terms of different clinical activity [18]. In particular, it would make it even more evident that the action of balneo-peloid therapy with M-peat is certainly not limited to the thermal effect, which is common to all peloids used with the bath technique [19].

The introduction in the thermal water bath of salty bromine–iodine water could favor and accelerate the microbial biodegradation of organic matter of plant origin that, as we know, is accompanied by the formation of humic acids [20].

In addition, in the muds after the maturation process, it is possible to observe the presence of proteins more in the deep layers than in the superficial ones. Consequently, it is possible to hypothesize the selective development of a microflora, essentially anaerobic, which is capable of migrating and moving deeply through the interstitial spaces of the maturing paste, with the adaptation of its metabolic/nutritional needs to the absence of light and the development of a heterotrophic type of nutrition, as previous studies have shown with diatoms [21,22].

Regarding the dosage of thiol groups, the poor results may be due to the absence of thiobacteria, typical of sulfur waters. As for the dosages carried out in the different samples, it should be noted that both for SH groups and for lipids, the greatest presence was found in the sample of peat matured for 6 months. It is reasonable to argue that longer periods of maturation do not determine particular advantages in relation to clinical activity. In this regard, it seems interesting to report the research of Cima et al. [23].

However, the antioxidant activity caused by the presence of SH groups could be indirect and could be through the action of humic acids not present in the mud. In fact, due to the characteristics of maturation in a confined environment, it is unlikely that the organic component can be traced back, as is already the case for other sludges, to humus degradation processes, i.e., to the microbial biodegradation of organic matter of vegetable or animal origin with the formation of humic acids (mostly expressed in natural mudpeat and sapropels) [21]. These substances are formed by the decomposition of plants, which generally happens naturally in water, in peat, and in soil, and in lignite (brown coal). The humic substances (HS) have a complex structure and can be fractionated into humin, humic acid, and fulvic acid.

### 4.2. Possible Therapeutic Effect of These Components

Both humic and fulvic acids exert antimicrobial activity in vitro. Antibacterial activities have been demonstrated against many bacteria including Staphylococcus aureus, Pseudomonas aeruginosa., Escherichia coli, Klebsiella pneumoniae as well as against Candida albicans [24,25].

Humic acid, oxyhumate (a water-soluble component of peat), and fulvic acid can also perform many other actions. In particular, oxyhumate has been shown to stimulate lymphocyte proliferation in HIV-positive subjects. Fulvic acid reduces the release of TNF-alpha at high concentrations [26]. HS possess antioxidant activity and stimulate the activation of nuclear factor Kappa B (NF-κB), an intracellular transcription factor that plays an important role during inflammation, especially in subjects affected by autoimmune diseases and during infections [27]. HS in particular protect the inflamed area by limiting the arrival of new inflammatory cells. HS’ mechanisms of action may also contribute to the inhibition of the two complement pathways (classical and alternative) as well as phagocyte degranulation and the production of related cytokines: IL-1β, IL-6, IL-10, and TNF-α [27].

There is a close correlation between HS and lipids, as reported in a study by Lethonen and coworkers [28], demonstrating that lipids in peat are structural units of humic acids. The oxidation of humic acids from highly decomposed peat, such as that in Lake Massaciuccoli, releases a significant number of lipids. Diterpenoids and unsaturated fatty acids are more present in the structure of humic acids than triterpenoids and saturated fatty acids, in addition to sterols. Diterpenes are compounds found primarily in terrestrial plants, algae, and fungi. Diterpenes have antimicrobial and anti-inflammatory properties. It is very likely that these acids derive from the age-old decomposition of conifer resins.

Therefore, it is not surprising that the analyses carried out with the Nile Red method on samples of M-peat revealed the presence of lipids. Furthermore, in a 1983 study, Agostini et al. showed the presence of palmitic acid, stearic acid, oleic acid, linoleic acid, and lipids with 22–24–26 carbon atoms in the M-peat [29]. The latter in particular are not present in mature peloids but only in peat. In a 2012 study [9], Rondanelli confirmed that unsaturated and saturated fatty acids are present in M-peat, including arachidonic acid, beenic acid (C22), and lignoceric acid (C24), as well as sterols such as ergosterol, sitosterol and cholesterol. Ergosterol is a fundamental component of the cell membrane of mycetes, where it performs the same functions as cholesterol in animal cells.

Arachidonic acid is also important; in fact, it is a substrate in several enzymatic reactions, including the cyclooxygenase (COX) pathway for the synthesis of prostaglandins and thromboxanes, and the lipoxygenase reaction for the synthesis of leukotrienes that exert multiple actions on the course of inflammatory processes. They are most likely co-responsible for the itching typical of some dermatological diseases (psoriasis, eczema, and acne) [30].

It is well known that lipids give a relevant contribution to the efficacy of peat for the treatment of dermatopathies and in wound care. It must be remembered that free fatty acids (FFA) are part of the lamellar membranes of the skin, in particular palmitic acid, stearic acid, beenic acid, lignoceric acid, and hexacosanoic acid (C26). All these FFA correspond to what was observed by Agostini and Rondanelli in the previously mentioned studies [9,29]. One must not forget oleic acid, which is less represented in M-peat, as well as linolenic and linoleic acid. These studies have also shown that although little is absorbed when they are applied directly to the skin, once penetrated, all these fatty acids certainly participate in the biochemical and metabolic processes that involve them [9,29].

The topical use of substances such as vegetable oils rich in saturated and unsaturated fatty acids can help us to better understand some of the activities of M-peat. Linolenic acid and linoleic acid, as well as oleic acid, can modulate surgical wound repair through the inhibition of nitric oxide production in the wound site. Linoleic acid shows a high chemotactic activity against macrophages which contribute to the autolytic cleaning of the wound, by increasing the production of metalloproteins so as to induce the process of granulation and thus accelerates the healing [31,32,33]. Finally, it should be stated that lipids are considered essential in the antioxidant activity of vegetable oils and therefore of M-peat that contains them: they are essential in maintaining the integrity of the epidermis aqueous barrier. Through this antioxidant action, a protective action against UV rays is also carried out, reducing the photo-oxidative ROS-induced injury that may lead to the degradation of collagen and its accumulation in the dermis, a process known as solar elastosis [34].

The strength of this study is the morphological and biochemical analysis performed, for the first time, on this peat. This is the first step towards a better understanding of the mechanisms of action of M-peat. Although our data constitute an interesting basis for potential developments in terms of research, the lack of characterization of the protein elements limits the possibilities for a better interpretation of the present results.

## 5. Conclusions

From the data shown in this study, it can be stated that the beneficial effects of M-peat are attributable to the antioxidant action that could be partly related to the presence of the identified SH groups. The confirmation of the presence of lipids allows a better understanding of some activities of M-peat, especially in dermatological applications.

In this work, it has been observed that the application of young mud (not yet mature) or decrepit mud (16-month-matured), also because of incorrect maturation due to the absence of stirring of the tanks, does not result in therapeutic effects; hence, there is interest in better defining the maturation time of M-peat and how it affects both the clinical activity and the cost/effectiveness ratio.

## Figures and Tables

**Table 1:** Analysis of the water used in the maturation of peat.

Ions	Mg/L
Na^+^	6500
K^+^	120
Mg^2+^	729
Ca^2+^	109
Sr^2+^	4.50
Al^2+^	0.61
Mn^2+^	0.56
Fe^2+^	0.83
Ba^2+^	0.32
HCO_3_^−^	307
SO_4_^2−^	1462
Cl^−^	12,400
Br^−^	56
SiO(OH)_3_	7.96

**Table 2:** Maturation period of peat and total protein, thiol protein, and lipid content.

		Total Proteins ^1^	Thiol Proteins ^1^	Total Lipids ^1^
	1	106	17.49	718.6
Basic peat	2	134	19.73	746.9
	3	118	18.18	769.5
	1	174	24.11	878.5
Peat matured for 6 months	2	170	26.19	965.0
	3	182	24.55	948.1
	1	121	18.55	847.5
Peat matured for 16 months	2	165	20.06	882.5
	3	139	18.18	872.5
	1	181	15.11	788.3
Peat matured for 36 months	2	174	18.12	870.1
	3	171	17.39	854.1

^1^ Total proteins, mg/mL; thiol proteins SH (µM); total lipids (A.U.).

## Data Availability

The data presented in this study are available on request from the corresponding author.
